# Supplementary material for: FGF8 promotes lipid droplet accumulation via the FGFR1/p-p38 axis in chondrocytes: FGF8 increases lipid droplet accumulation
Source: Acta Biochim Biophys Sin (Shanghai). 2025 May 15;58(2):258–74. doi: 10.3724/abbs.2025075 (PMC12900779; doi:10.3724/abbs.2025075)

**Supplementary Figures**

**
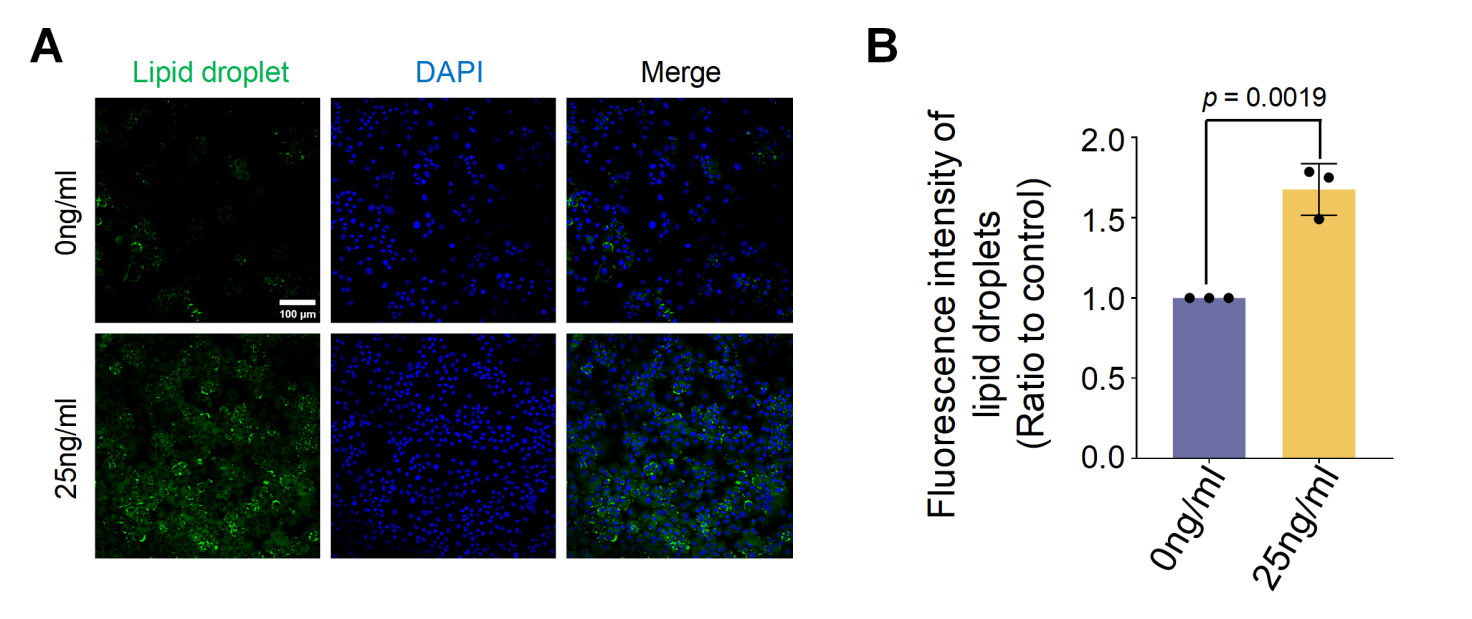
**

**Supplementary Figure S1. FGF8 promotes lipid droplet accumulation in chondrocytes** (A) Representative fluorescence images (40×) showing lipid droplet accumulation in chondrocytes induced by FGF8 at 25 ng/mL for 2 days. (B) Total fluorescence quantification showing the change of lipid droplets in chondrocytes induced by FGF8 at 25 ng/mL for 2 days in (A). The data are based on 3 independent replicates (*n* = 3). The data in (B) are based on two-tailed Student’s *t* test. The data in (B) are presented as the mean ± SD. *P* < 0.05.


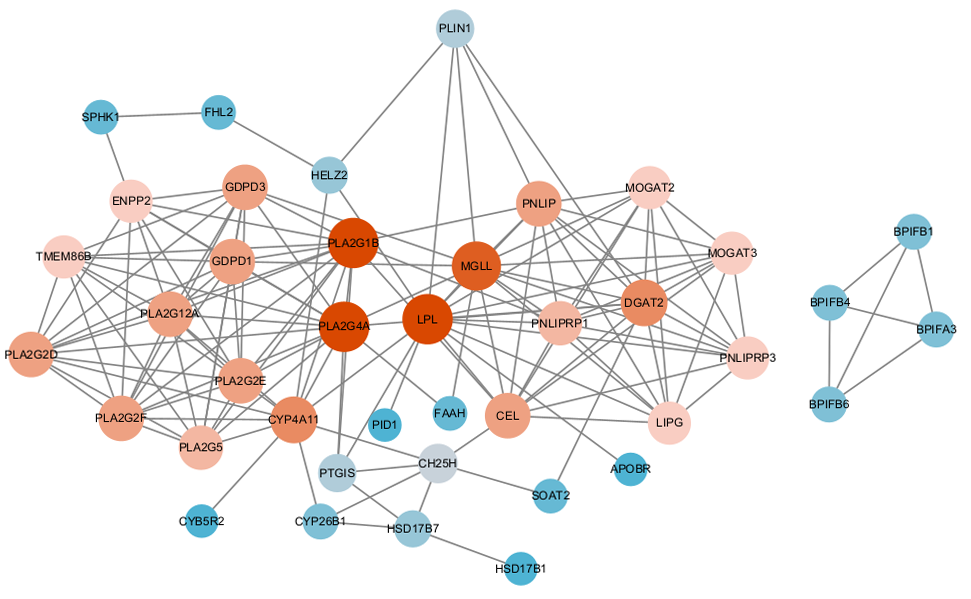


**Supplementary Figure S2. A protein-protein interaction analysis using STRING confirms that FGF8-mediated lipid accumulation involves a series of regulatory proteins** This figure is related to **Figure 2A**.


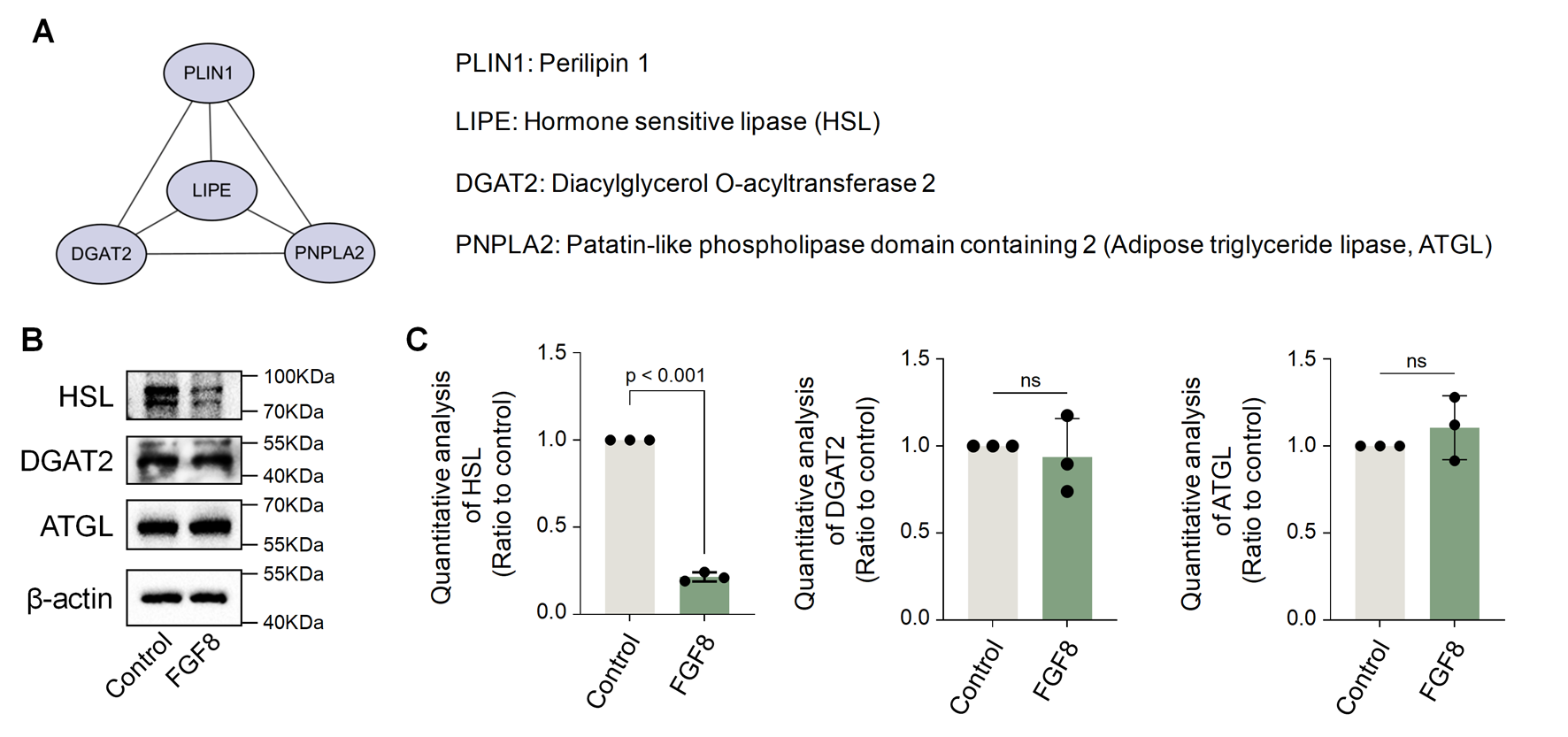


**Supplementary Figure S3. The expressions of Plin1 partner proteins in chondrocytes induced by FGF8** (A) A protein-protein interaction analysis by STRING confirmed the relationship between Plin1 and the partner proteins. (B) Western blot analysis showing changes in HSL, DGAT2, and ATGL in chondrocytes induced by FGF8. (C) Quantitative analysis of HSL, DGAT2, and ATGL in (B). The data are based on 3 independent results (*n* = 3).

**
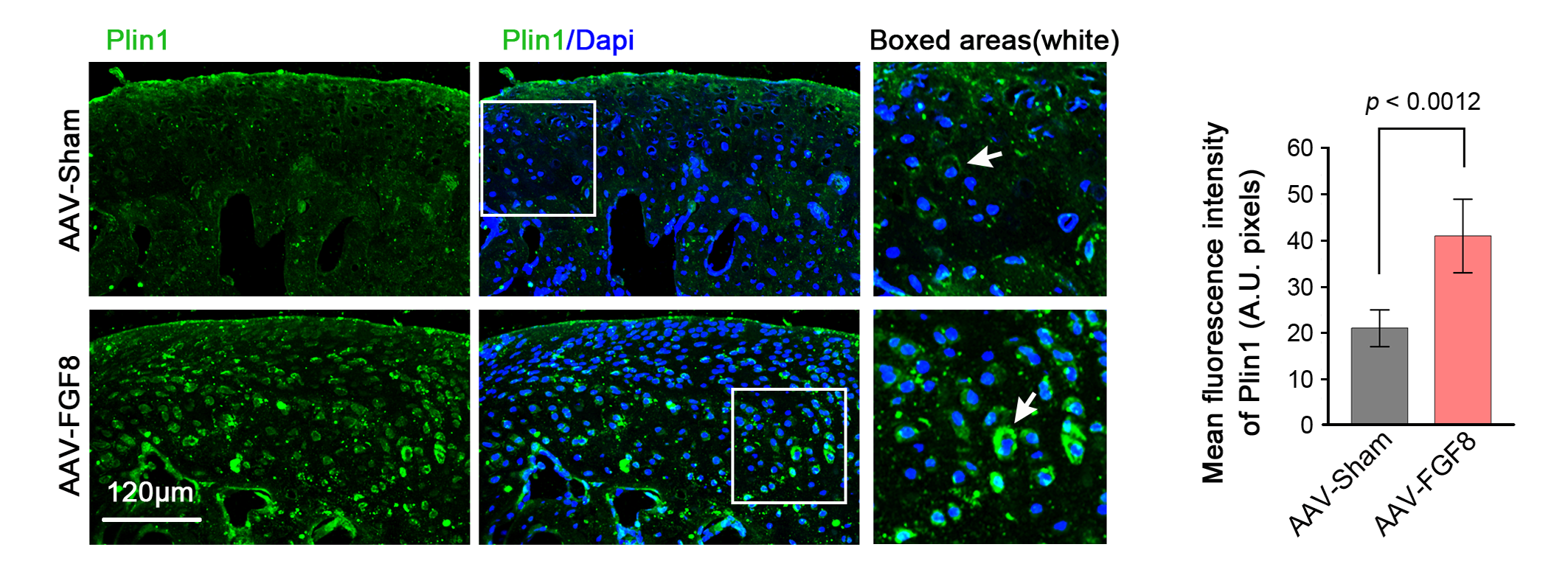
**

**Supplementary Figure S4. FGF8 induces a higher expression of Plin1 in cartilage tissue** The white arrows indicate the representative expressions of Plin1 in chondrocytes. This figure is related to the **Figure 2C**.


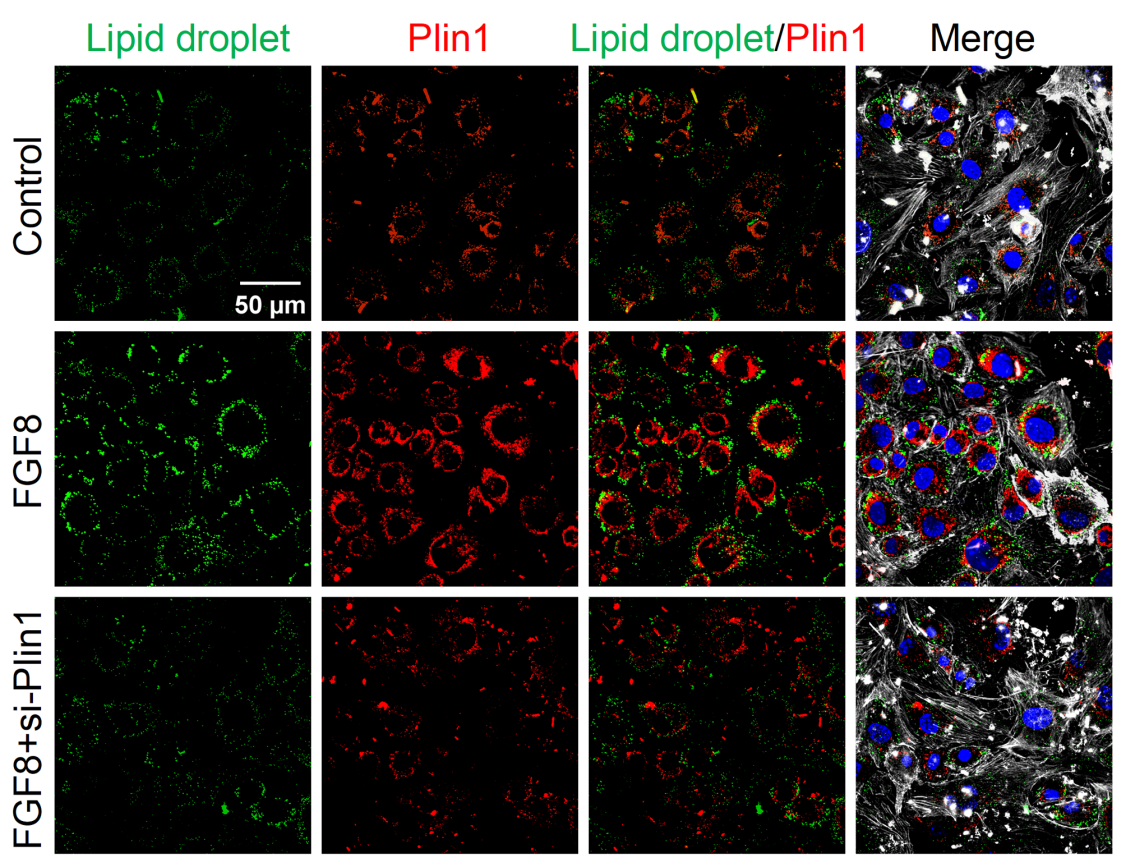


**Supplementary Figure S5. FGF8 promotes lipid droplet accumulation and plin1 expression in chondrocytes** Representative fluorescence images (60×) of multiple cells showing the changes of lipid droplets and plin1 in chondrocytes induced by si-plin1 in the presence of FGF8.

**
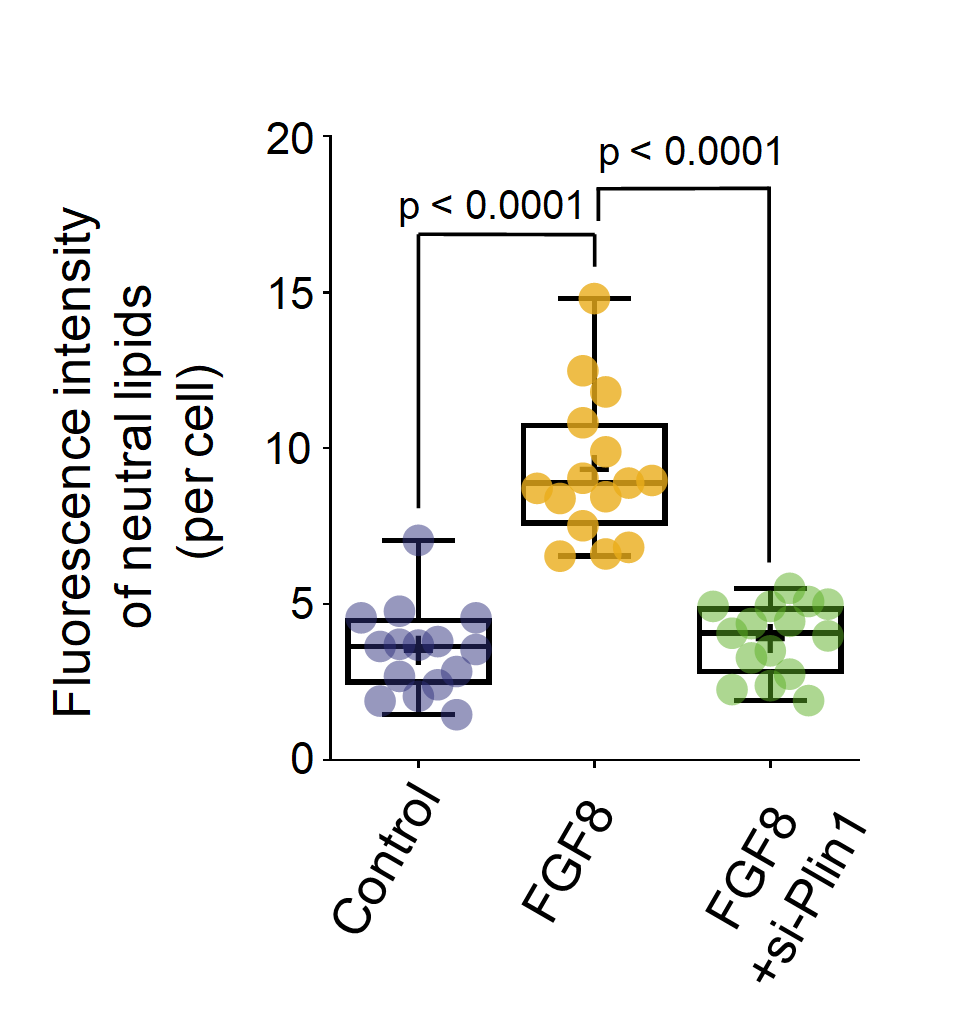
**

**Supplementary Figure S6. Total fluorescence quantification of lipids per cell by si-Plin1 in the presence of FGF8** The data are based on 15 cells from 3 independent samples (*n* = 3). This figure is related to **figure 2F**. The data are shown in the box (from 25%, 50% to 75%) and whisker (minimum to maximum) plots. The statistic analysis is based on one-way analysis of variance and differences are considered significant at *P* < 0.05.

**
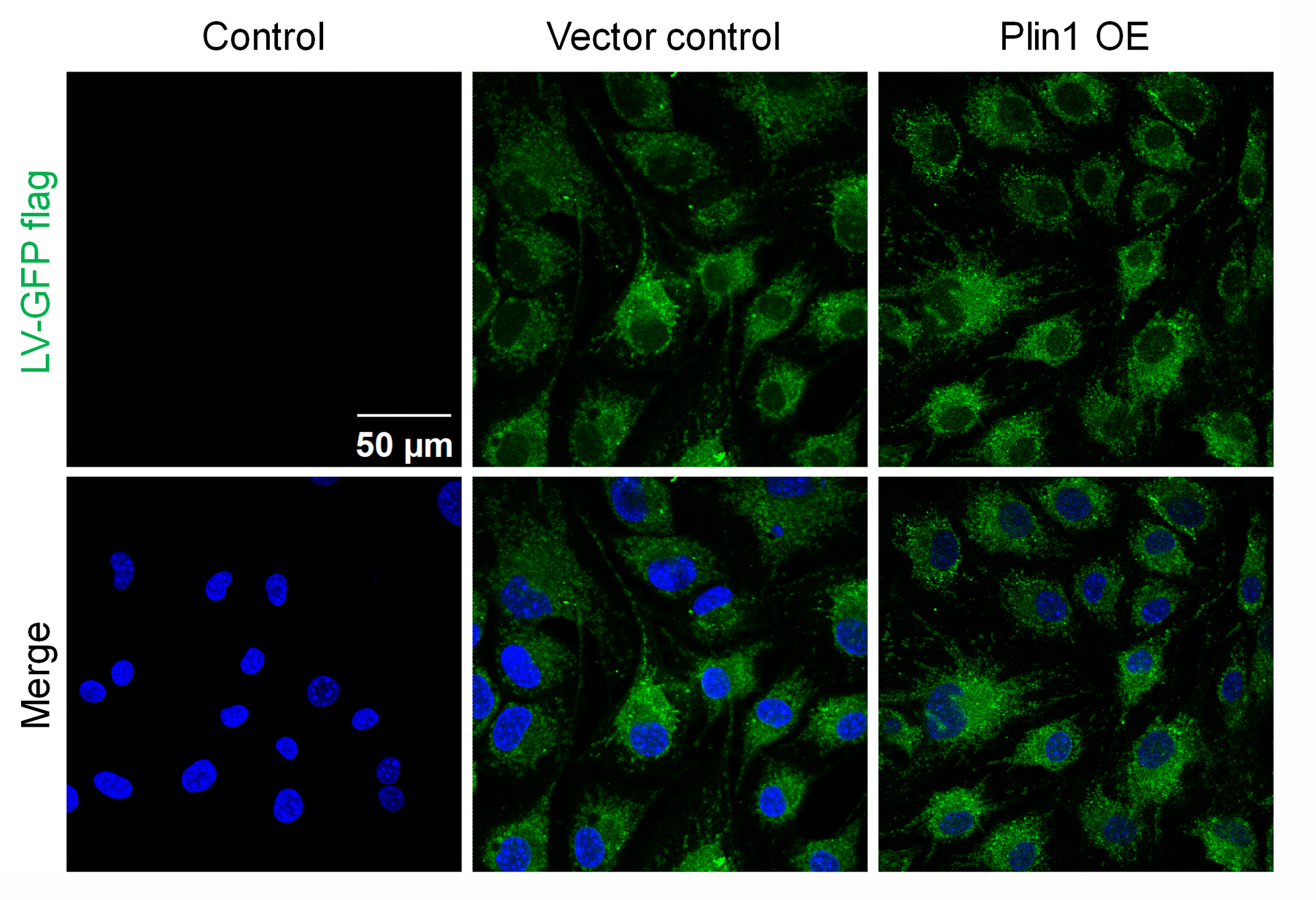
**

**Supplementary Figure S7.** **The transfection efficiency of lentivirus carrying *Plin1* gene in chondrocytes** Representative fluorescence images (60×) of multiple cells showing the efficiency of lentivirus transfection in chondrocytes at 30 MOI (multiplicity of infection). Green fluorescence indicates GFP protein carried by lentivirus and blue fluorescence indicates the nuclei.

**
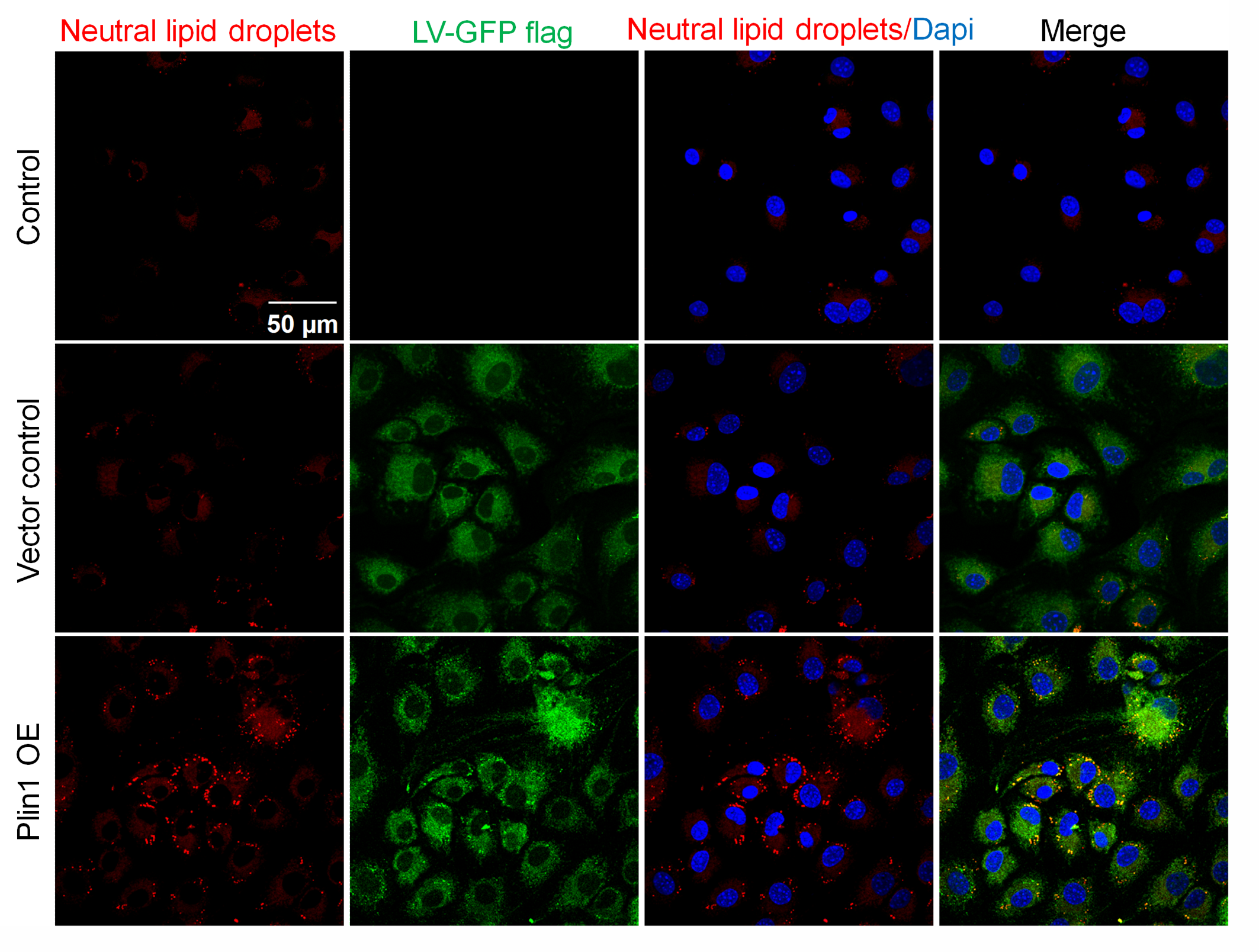
**

**Supplementary Figure S8. Overexpression of Plin1 promotes lipid droplet accumulation in chondrocytes** Representative fluorescence images (60×) of multiple cells showing the changes of lipid droplet accumulation in chondrocytes induced by overexpressing Plin1.

**
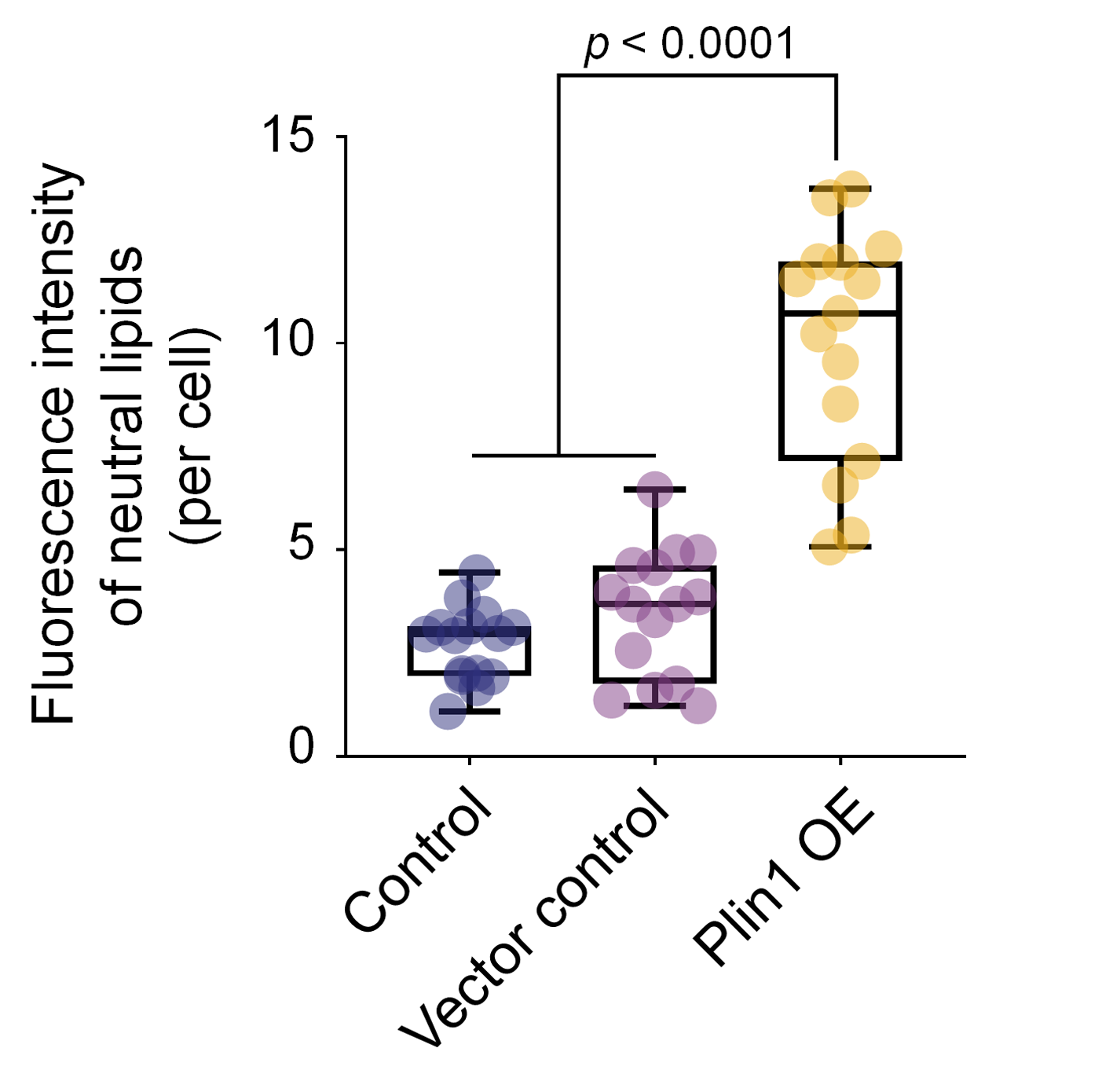
**

**Supplementary Figure S9. Total fluorescence quantification of lipids per cell by overexpression of Plin1** The data are based on 15 cells from 3 independent samples (*n* = 3). This figure is related to **Figure 2P**. The data are shown in the box (from 25%, 50% to 75%) and whisker (minimum to maximum) plots. The statistic analysis is based on two-tailed Student’s *t* test and differences are considered significant at *P* < 0.05.


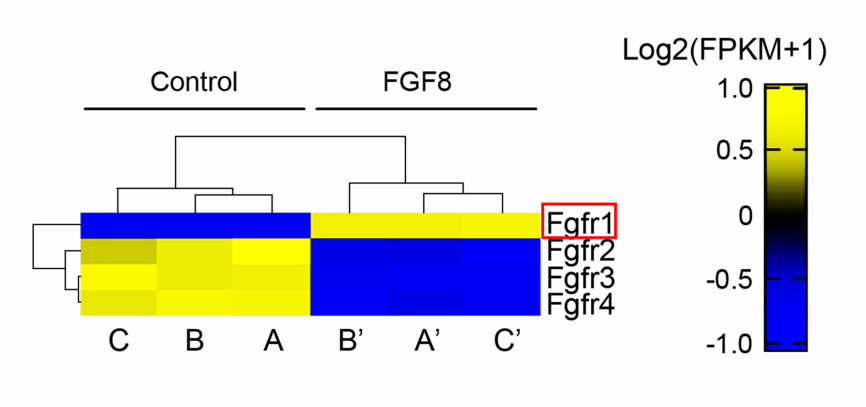


**Supplementary Figure S10. The gene expression of FGFRs in chondrocytes induced by FGF8** The pheatmap based on RNA sequencing showing the gene changes of FGFRs in chondrocytes induced by FGF8 at 25 ng/mL for 24 h.

**
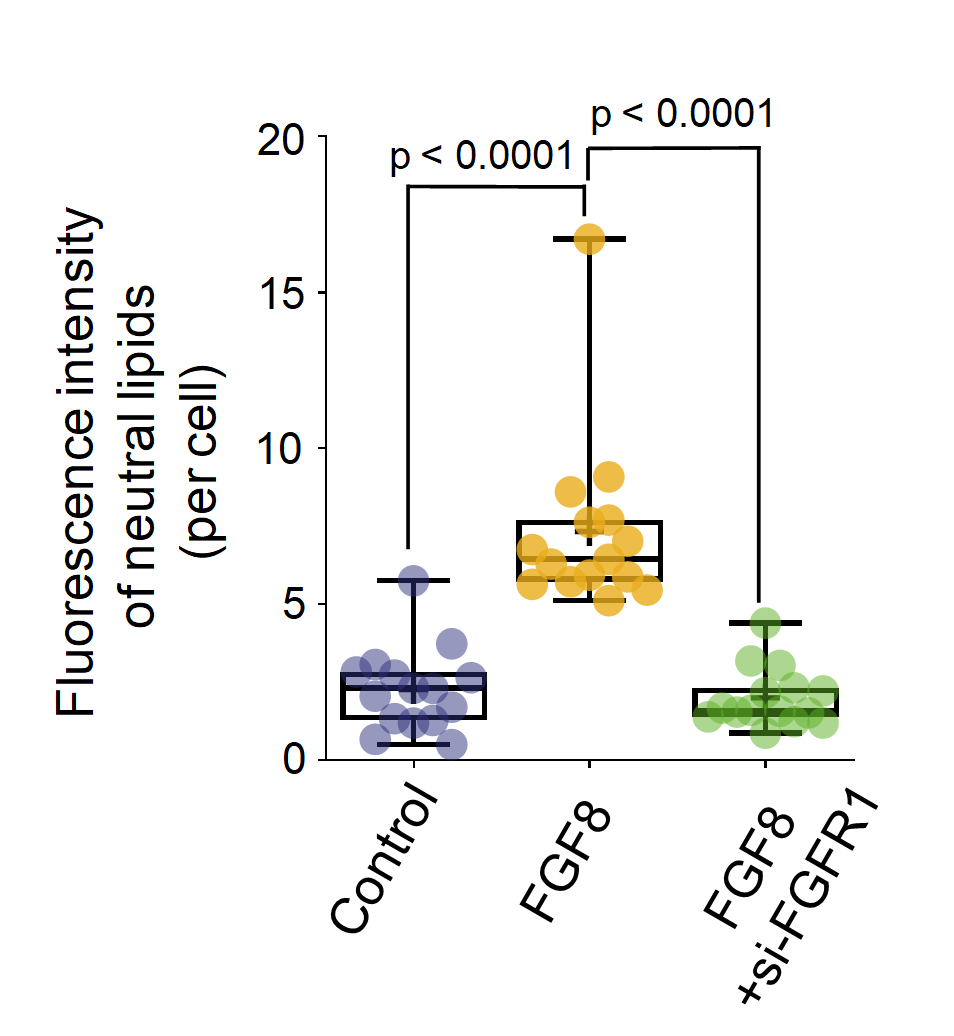
**

**Supplementary Figure S11. Total fluorescence quantification of lipids per cell by si-FGFR1 in the presence of FGF8** The data are based on 15 cells from 3 independent samples (*n* = 3). This figure is related to **Figure 3G**. The data are shown in the box (from 25%, 50% to 75%) and whisker (minimum to maximum) plots. The statistic analysis is based on one-way analysis of variance and differences are considered significant at *P* < 0.05.

**
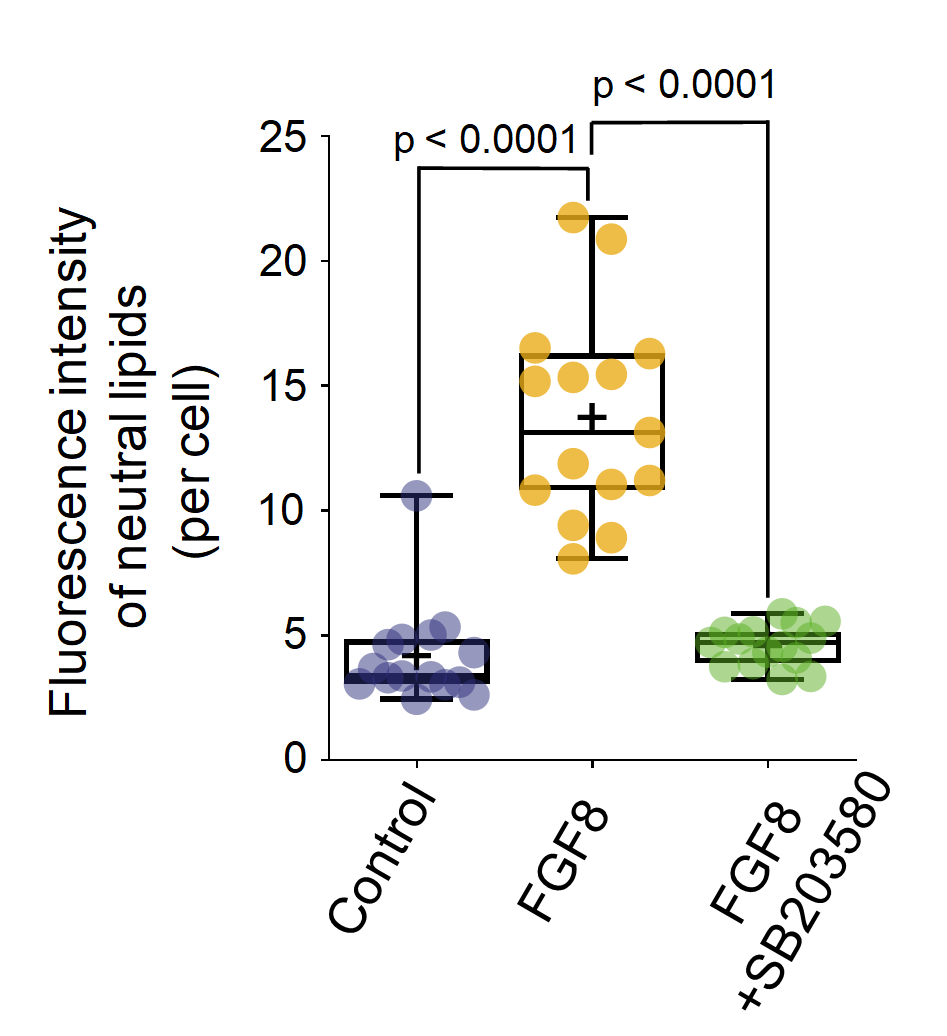
**

**Figure S12. Total fluorescence quantification of lipids per cell treated with SB203580 in the presence of FGF8** The data are based on 15 cells from 3 independent samples (*n* = 3). This figure is related to **Figure 5I**. The data are shown in the box (from 25%, 50% to 75%) and whisker (minimum to maximum) plots. The statistic analysis is based on one-way analysis of variance and differences are considered significant at *P* < 0.05.

**Supplementary Tables**

**Supplementary Table S1. KEGG analysis showing upregulated pathways in chondrocytes induced by FGF8**

**Supplementary** **Table S1. KEGG analysis showing downregulated pathways in chondrocytes induced by FGF8**

**Supplementary** **Table S2. Original data for candidate mediators involved in lipid droplet accumulation**

**Supplementary** **Table S3. The details for lentivirus information (Hanbio, Shanghai, China)**

**Gene sequence**

**5**′**-**ATGTCAATGAACAAGGGCCCAACCCTGCTGGATGGAGACCTCCCTGAGCAGGAGAACGTGCTCCAGAGAGTTCTGCAGCTGCCTGTGGTGAGCGGGACCTGTGAGTGCTTCCAGAAGACCTACAACAGCACCAAAGAAGCCCACCCCCTGGTGGCCTCTGTGTGCAATGCCTATGAGAAGGGTGTACAGGGTGCCAGCAACCTGGCTGCCTGGAGCATGGAGCCGGTGGTCCGTCGGCTGTCCACCCAGTTCACAGCTGCCAATGAGTTGGCCTGCAGAGGCCTGGACCACCTGGAGGAAAAGATCCCGGCTCTTCAATACCCTCCAGAAAAGATCGCCTCTGAACTGAAGGGCACCATCTCTACCCGCCTTCGAAGCGCCAGGAACAGCATCAGTGTGCCCATTGCAAGCACCTCTGACAAGGTTCTGGGGGCCACTCTGGCCGGCTGCGAGCTTGCCTTGGGGATGGCCAAAGAGACAGCAGAATATGCCGCCAACACCCGGGTTGGCCGACTGGCCTCTGGAGGGGCTGATCTGGCTCTGGGAAGCATCGAGAAGGTGGTAGAGTTCCTCCTGCCACCAGACAAGGAGTCAGCCCCTTCTTCCGGACGGCAGAGGACCCAGAAGGCTCCCAAGGCCAAACCAAGCCTTGTGAGGAGGGTCAGCACCCTGGCCAACACTCTTTCTCGACACACCATGCAAACCACAGCATGGGCCCTGAAGCAGGGCCACTCTCTGGCCATGTGGATCCCGGGTGTGGCACCCCTGAGCAGCCTGGCCCAGTGGGGCGCATCGGCAGCCATGCAGGTGGTGTCCCGGCGGCAGAGTGAGGTGCGGGTGCCCTGGCTGCACAACCTGGCAGCCTCTCAGGATGAGAGCCATGACGACCAGACAGACACAGAGGGAGAGGAGACAGACGACGAGGAGGAGGAAGAAGAGTCCGAGGCTGAGGAGAACGTGCTCAGAGAGGTTACAGCCCTGCCCAACCCGAGAGGCCTCCTGGGTGGTGTGGTACACACCGTGCAGAACACTCTCCGGAACACCATCTCCGCAGTGACCTGGGCACCTGCGGCTGTGCTGGGCACGGTGGGAAGGATCCTGCACCTCACACCAGCCCAGGCTGTCTCCTCTACCAAAGGGAGGGCCATGTCCCTATCCGATGCCCTGAAGGGTGTTACGGATAACGTGGTAGACACTGTGGTACACTATGTGCCGCTTCCCAGGCTGTCCCTGATGGAGCCCGAGAGCGAATTCCGAGACATCGATAACCCTTCAGCAGAGGCGGAGCGCAAAGGGTCCGGGGCGCGGCCCGCCAGCCCGGAGTCCACCCCGCGCCCGGGCCAGCCCCGCGGCAGCTTGCGCAGCGTGCGGGGTCTCAGCGCGCCCTCCTGCCCCGGCCTGGACGACAAAACCGAGGCGTCAGCGCGTCCCGGCTTCCTGGCTATGCCCAGAGAGAAGCCTGCGCGCAGAGTCAGCGACAGCTTCTTCCGGCCCAGCGTCATGGAGCCCATCCTGGGCCGCGCGCAGTACAGCCAGCTGCGCAAGAAGAGCTGA-**3**′

**3. Original images of western blots**


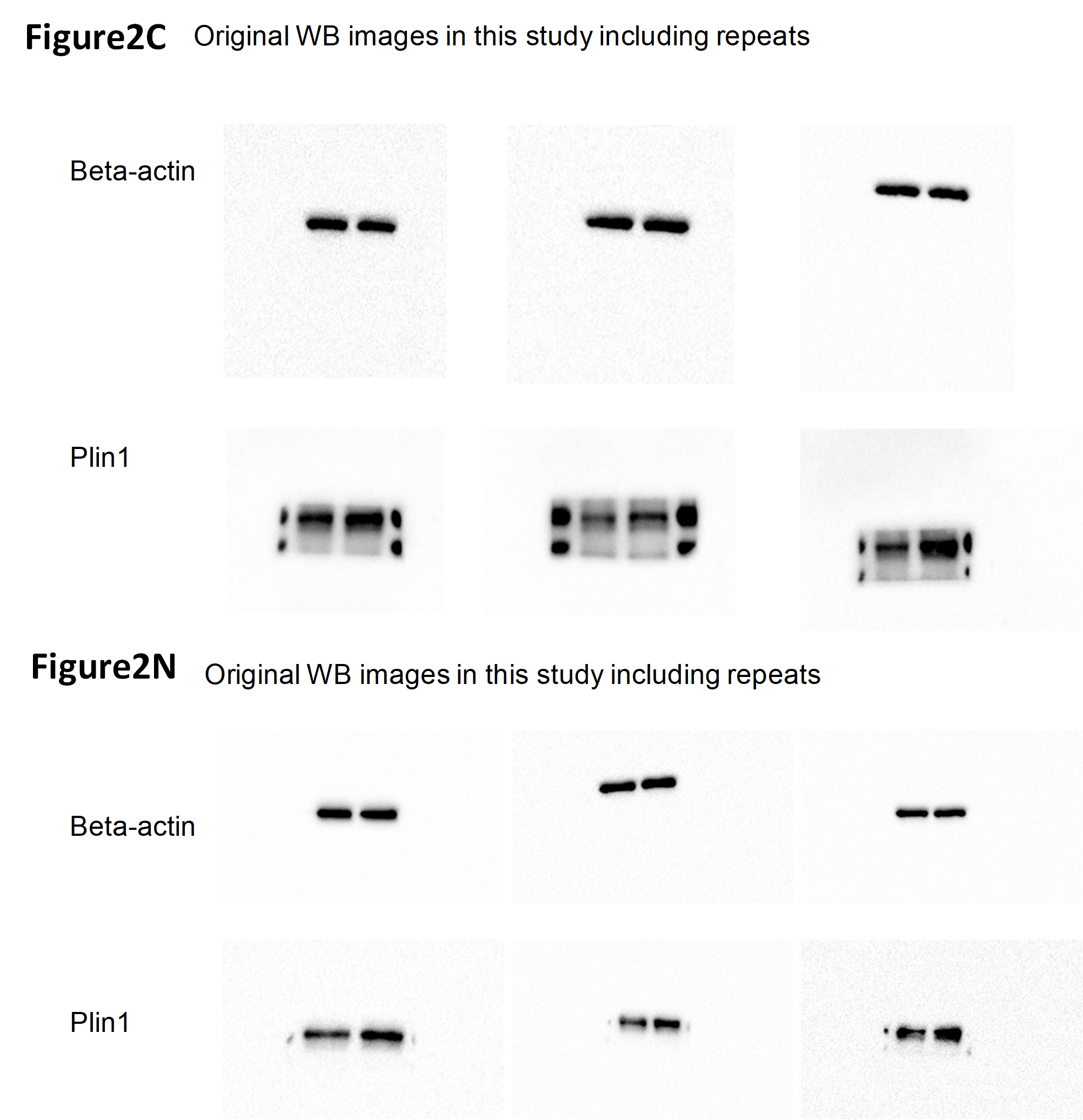


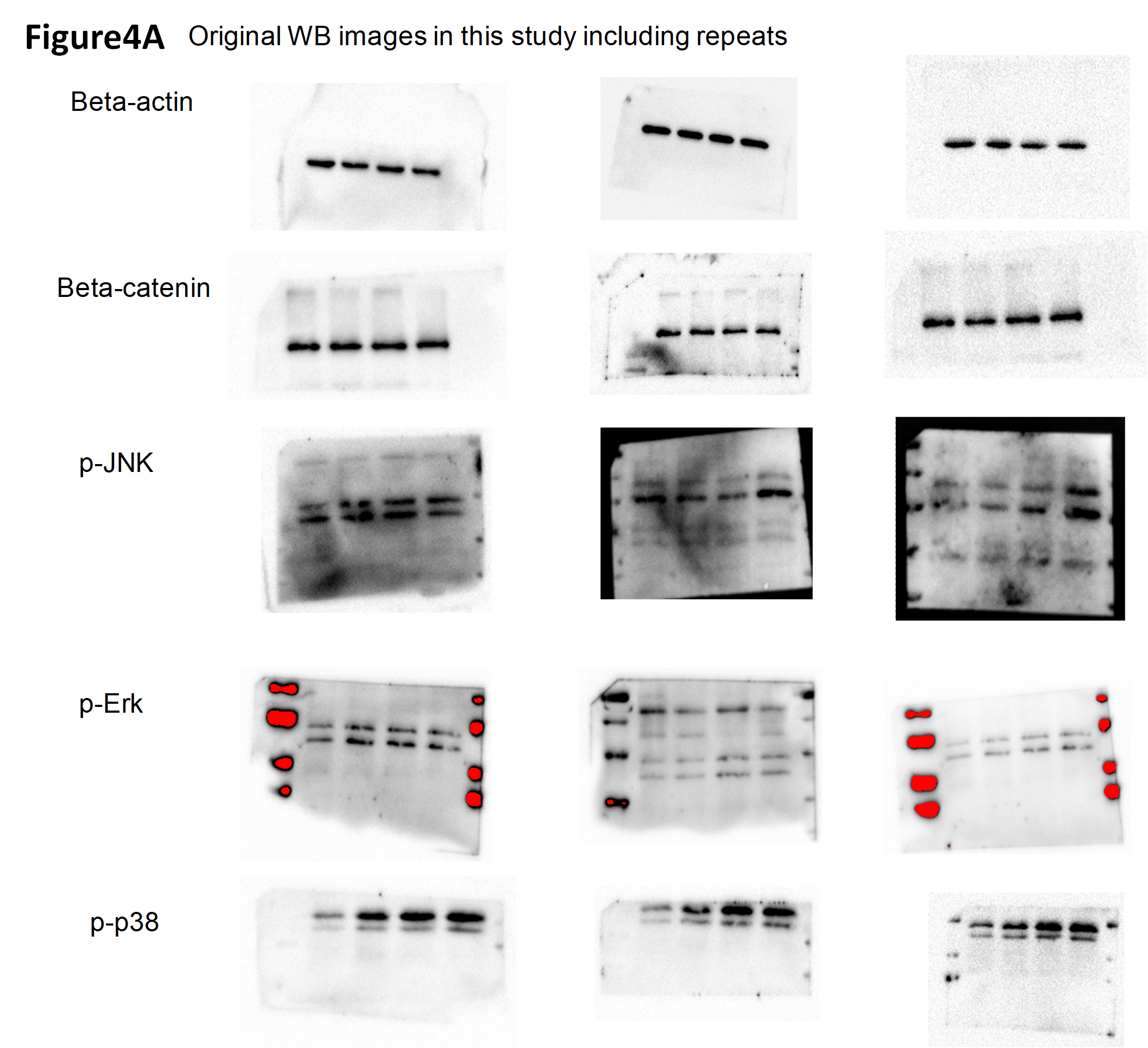


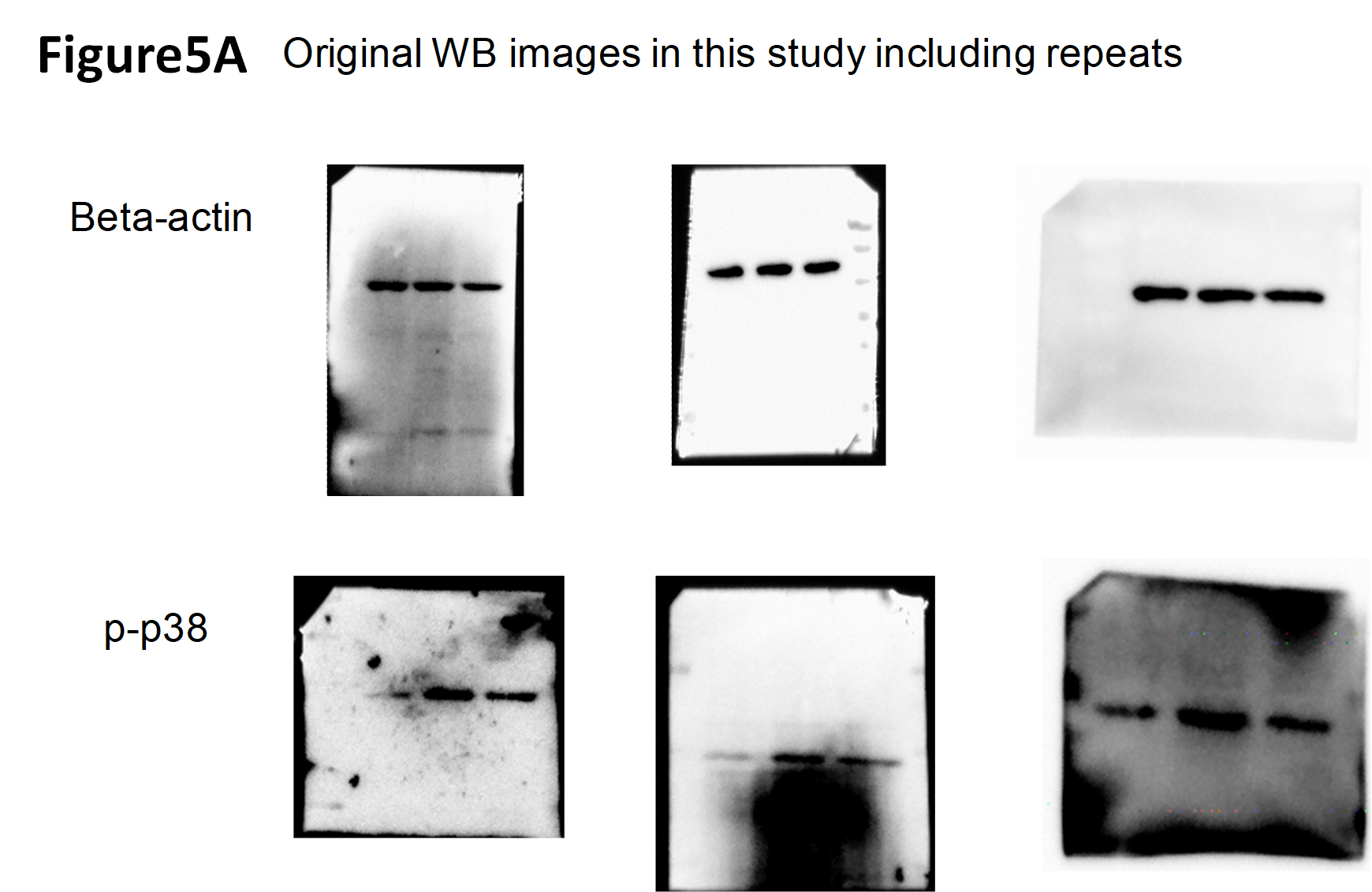


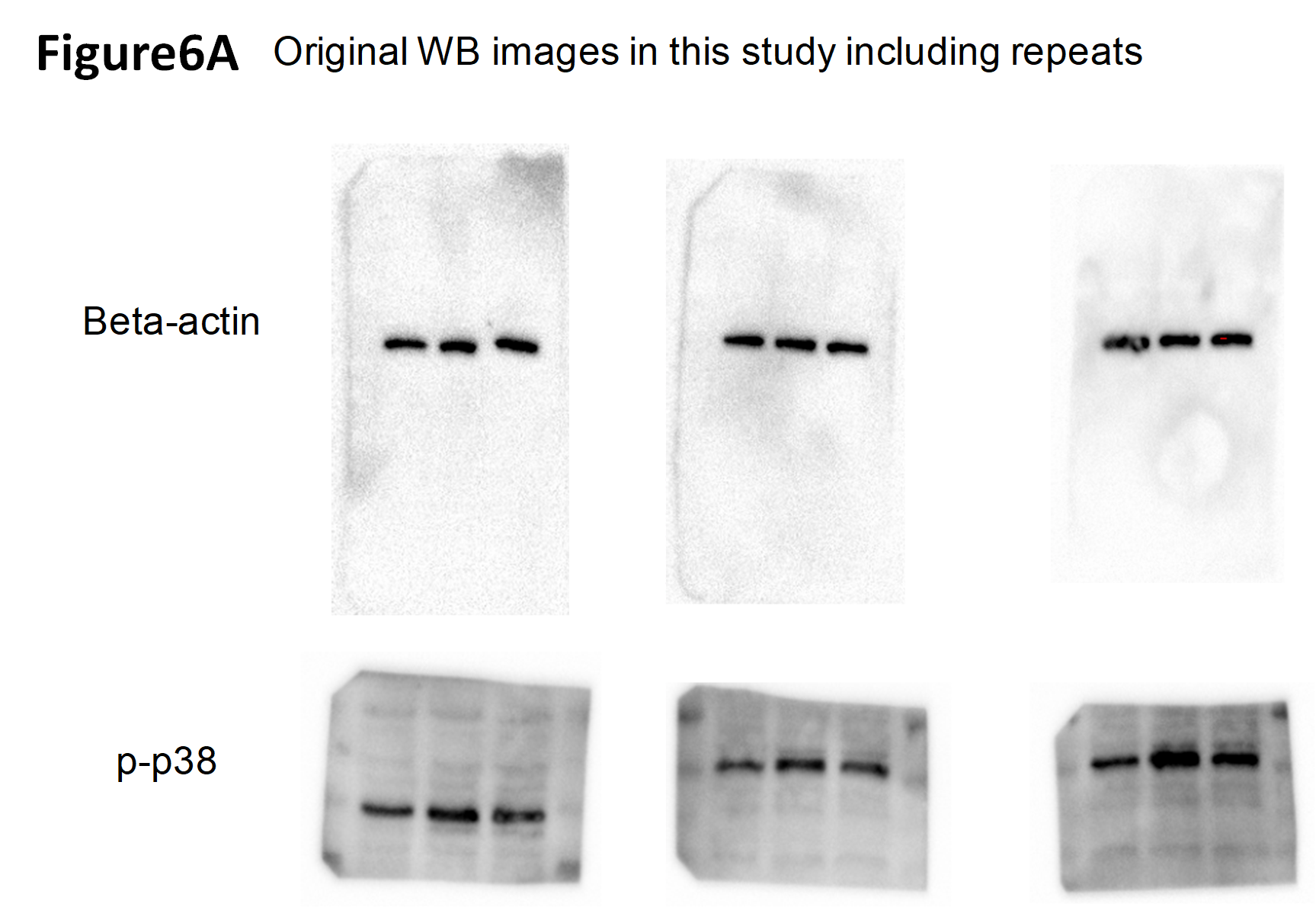

Supplement: 25132Supplementary_Data [file 25132Supplementary_Data.doc]
